# Microbiome and Culture Based Analysis of Chronic Rhinosinusitis Compared to Healthy Sinus Mucosa

**DOI:** 10.3389/fmicb.2018.00643

**Published:** 2018-04-17

**Authors:** Kerstin Koeller, Daniel P. R. Herlemann, Tobias Schuldt, Attila Ovari, Ellen Guder, Andreas Podbielski, Bernd Kreikemeyer, Bernhard Olzowy

**Affiliations:** ^1^Institute of Medical Microbiology, Virology and Hygiene, University Medicine Rostock, Rostock, Germany; ^2^Biological Oceanography Section, Leibniz Institute for Baltic Sea Research, Warnemünde, Rostock, Germany; ^3^Center of Limnology, Estonian University of Life Sciences, Tartu, Estonia; ^4^Department of Otorhinolaryngology, Head and Neck Surgery, University Medicine Rostock, Rostock, Germany; ^5^HNO-Zentrum Landsberg, Landsberg am Lech, Germany; ^6^Department of Otorhinolaryngology, Head and Neck Surgery, University of Munich Medical Center, Munich, Germany

**Keywords:** chronic rhinosinusitis, microbiology, 16S rRNA gene sequencing, community analysis, microbiome

## Abstract

The role of bacteria in chronic rhinosinusitis (CRS) is still not well understood. Whole microbiome analysis adds new aspects to our current understanding that is mainly based on isolated bacteria. It is still unclear how the results of microbiome analysis and the classical culture based approaches interrelate. To address this, middle meatus swabs and tissue samples were obtained during sinus surgery in 5 patients with CRS with nasal polyps (CRSwNP), 5 patients with diffuse CRS without nasal polyps (CRSsNP), 5 patients with unilateral purulent maxillary CRS (upm CRS) and 3 patients with healthy sinus mucosa. Swabs were cultured, and associated bacteria were identified. Additionally, parts of each tissue sample also underwent culture approaches, and in parallel DNA was extracted for 16S rRNA gene amplicon-based microbiome analysis. From tissue samples 4.2 ± 1.2 distinct species per patient were cultured, from swabs 5.4 ± 1.6. The most frequently cultured species from the swabs were *Propionibacterium acnes, Staphylococcus epidermidis, Corynebacterium* spp. and *Staphylococcus aureus*. The 16S-RNA gene analysis revealed no clear differentiation of the bacterial community of healthy compared to CRS samples of unilateral purulent maxillary CRS and CRSwNP. However, the bacterial community of CRSsNP differed significantly from the healthy controls. In the CRSsNP samples *Flavobacterium, Pseudomonas, Pedobacter, Porphyromonas, Stenotrophomonas*, and *Brevundimonas* were significantly enriched compared to the healthy controls. Species isolated from culture did not generally correspond with the most abundant genera in microbiome analysis. Only *Fusobacteria, Parvimonas*, and *Prevotella* found in 2 unilateral purulent maxillary CRS samples by the cultivation dependent approach were also found in the cultivation independent approach in high abundance, suggesting a classic infectious pathogenesis of odontogenic origin in these two specific cases. Alterations of the bacterial community might be a more crucial factor for the development of CRSsNP compared to CRSwNP. Further studies are needed to investigate the relation between bacterial community characteristics and the development of CRSsNP.

## Introduction

Chronic rhinosinusitis (CRS) is among the most prevalent of all chronic diseases and affects 5–15% of the general population in Europe and the USA. According to the European Position Paper on Rhinosinusitis and Nasal Polyps 2012, CRS is diagnosed clinically when a patient suffers two or more symptoms out of nasal blockage, nasal discharge, loss of smell and facial pressure or pain for more than 3 months with an additional finding of endoscopic signs of inflammation in the region of the middle meatus (swelling, polyps, mucopurulent discharge) and/or mucosal alterations in the computed tomography scan. This complex of symptoms can originate from sinunasal mucosal inflammation as a result of various conditions. CRS with nasal polyps (CRSwNP) and CRS without nasal polyps (CRSsNP) are the two major clinical phenotypes that also imply different therapeutic approaches with CRSwNP being more responsive to therapy with corticosteroids (Fokkens et al., 2012). CRS is certainly not a classic infectious disease, but has a complex, multifactorial etiology. Ostiomeatal complex obstruction has long been deemed a major factor, but its actual role might have been overestimated and seems to be more important in CRS without nasal polyps (CRSsNP) compared to CRS with nasal polyps (CRSwNP) (Chandra et al., 2010; Leung et al., 2011). More recent concepts see the chronic mucosal inflammation result from an inappropriate or excessive immune response to environmental factors such as bacteria, fungi or cigarette smoke (Kern et al., 2008; Hoggard et al., 2017). Current research mainly addresses the pathologic host response itself and the external factors with bacteria being the main focus (Hoggard et al., 2017).

The introduction of culture-independent bacterial gene sequencing techniques into the study of CRS in the past few years has significantly altered current thinking on the role of bacteria in the disease process (Ramakrishnan et al., 2016; Hoggard et al., 2017). Healthy sinus mucosa harbors rich and diverse bacterial populations while the diseased state is characterized by a reduced bacterial richness (Abreu et al., 2012), but not an increase in the total number of bacteria (Ramakrishnan et al., 2015). Healthy sinus mucosa is frequently colonized by small numbers of pathogenic bacteria and *S. aureus* can even be found as abundant species without causing symptoms (Ramakrishnan et al., 2016).

These observations challenge the role of antibiotics in the treatment of CRS and their use might even be a relevant risk factor for the development of CRS (Maxfield et al., 2016). Antibiotic guidelines suggest antibiotic therapy guided by bacterial culture for acute exacerbation of CRS (Federspil et al., 2009; Olzowy et al., 2018). However, bacterial culture only reveals a small subset of bacteria detectable by culture independent broad-range analysis of 16S rRNA (Feazel et al., 2012) and the clinical relevance of this subset seems largely unclear. The aim of this study was to examine how the clinically available culture techniques interrelate with the microbiome analyzed via 16S rRNA gene high throughput sequencing and to evaluate the bacterial community composition on the distinct types of CRS compared to healthy bacterial communities.

## Materials and methods

### Study design

The study protocol was approved by the local ethics committee under the Reg.-Nr. A 2012-0142. Patients were recruited in 2013 at the Department of Otorhinolaryngology, Head and Neck Surgery, University Medicine Rostock, Germany. CRS was defined according to the European Position Paper on Rhinosinusitis and Nasal Polyps 2012 (Fokkens et al., 2012). An equal number of patients scheduled for sinus surgery for CRSwNP, CRSsNP, and unilateral purulent maxillary CRS were recruited. Selection of the patients was based on the pre- and intra-operative endoscopic findings. For the CRSsNP group, patients with signs of mucosal edema were excluded, to avoid including early-stage CRSwNP patients; for the CRSwNP group only patients with evident polypoid masses were included. Patients without a history of sinus complaints who received sinus surgery for orbital decompression, an orbital tumor and a posttraumatic mucocele served as control. Patients were excluded when they had received antibiotics 4 weeks prior to surgery. Written informed consent was obtained from all patients the day before surgery.

### Sampling

Intraoperatively the anterior nares were cleaned with iodine solution and two swabs, flocked and not flocked with the aim to enlarge the probability to get all microorganisms present, were taken from the middle meatus of one side under endoscopic control. In a second step, again flocked and not flocked swabs were taken intraoperatively after the anterior ethmoid and the maxillary sinus had been opened. Care was taken to avoid contamination from other mucosal sites. In addition, three mucosal biopsies were obtained of each patient from the same side. In primary cases the uncinate process, the bulla ethmoidalis and a part of the medial wall of the maxillary sinus posterior to the natural ostium were excised with cutting instruments. In revision cases of nasal polyps three larger pieces were taken, one from the first accessible polyps in the nasal cavity and two from deeper regions out of the maxillary sinus, the frontal recess or the sphenoid sinus. The remaining tissue that was removed during the subsequent sinus surgery was subjected to Hematoxin-Eosin staining and evaluated for the proportion of eosinophilic granulocytes among the inflammatory cell infiltrate.

### Microbiological diagnostic procedures

Within 1 h from sampling, materials were sent in a styrofoam transport container to the laboratory and culturing was promptly started, so that maximum duration between sampling and culturing was 2 h. Colonies were obtained by streaking swabs in a standardized fashion onto Colombia agar supplemented with 5% sheep blood, chocolate agar and Schaedler agar in 3 streaks with a length of 5 cm while constantly rotating the swab shaft in an angle of 45° to the plate and exerting gentle pressure. Subsequently, swabs were rotated first in brain heart infusion medium (BHI, Oxoid) and finally for anaerobic incubation conditions in prereduced thioglycollate (Sifin, Berlin) broth supplemented with hemin (Serva) and vitamin K (Fluka) (10% CO_2_/10% H_2_/80% N_2_).

Biopsies were washed three times with phosphate buffered saline (PBS, pH 7.4) before cutting them into small pieces. Two pieces were frozen at −20°C for microbiome analysis and fixed for 24 h in a solution containing 2.5% glutardialdehyde, respectively. Remaining pieces were macerated and used for inoculation of agar plates as well as BHI and thioglycollate broth.

Colombia and chocolate agar plates were subsequently cultured at 37°C under a 5% CO_2_ – 20% O_2_ atmosphere while Schaedler agar plates were cultured at 37°C under an anaerobic atmosphere (10% CO_2_/10% H_2_/80% N_2_). BHI and thioglycollate broth were inspected daily and in case of visible bacterial growth (turbidity of the broth) subcultured on Colombia, chocolate and Schaedler agar plates with subsequent aerobic and anaerobic incubation, respectively.

Colonies were differentiated by color, form, hemolysis, consistency and were counted semiquantitatively by macroscopic inspection. Gram-staining, potassium hydroxide-, cytochrome-oxidase, and catalase-testing were used for further differentiation. Finally, all types of colonies were identified using matrix-assisted laser-desorption-ionization time-of-flight mass spectrometry (MALDI-TOF-MS) using a Shimadzu “AXIMA Assurance” MALDI-TOF mass spectrometer (Shimadzu, Germany Ltd., Duisburg, Germany) as described elsewhere (Frickmann et al., 2013), VITEK® 2 system (bioMérieux, Marcy l'Etoile, France), agglutination assay (Slidex Staph Pluis, bioMérieux, Marcy l'Etoile, France) and Pastorex™ Strep A-B-C-D-F-G Test, (BioRad Laboratories GmbH, München, German), respectively, apiweb™-system (bioMérieux, Marcy l'Etoile, France), RapID™ Systems (Thermo Fisher Scientific, Remel Products, Lenexa, KS) or 16S rDNA sequencing.

Viridans streptococci and coryneform bacteria were not further sub-classified.

### DNA isolation, library preparation and sequencing

Whole genomic DNA was isolated from the patient tissue samples using the “DNeasy Blood and Tissue Kit from Qiagen,” basically following the instructions of the manual. The concentration of purified DNA was determined by Qubit and Nanodrop protocols and finally amplicon PCR was started with DNA templates at a concentration of 5 ng/μl in 10 mM Tris pH 8.5. The used primers targeted the V3/V4 region of the 16S rRNA encoding gene and were selected from Herlemann et al. (2011). This resulted in amplicon sizes of roughly 450 bp. All further steps in library preparation were performed according to the Illumina “16S Metagenomic Sequencing Library Preparation” protocol.

Briefly, PCR clean-up, Index PCR, PCR clean-up 2, library quantification, normalization and pooling were performed according to the above referred manual. Bioanalyzer DNA 1000 chips (Agilent Technologies) and Qubit kits (Thermo Fischer Scientific) were used for quantity and quality controls of each individual sample library and the final library pool. Ten percent PhiX control was spiked into the final pool. 4 pM of the final library pool was subjected to one individual sequencing run using a 500 cycle V2 chemistry kit on an Illumina MiSeq machine. During the run roughly 1,000 (K/mm^2^) clusters were sequenced, generating ca. 15 million reads passing filter specs. Over 75% of the sequencing and index reads were found with a Qscore ≥30. All raw data fastq files were recovered from the machine and used for further sequence data processing as outlined below.

### Data processing/analysis

A total of 11,096,494 reads with an average length of 242 bp (from the forward primer) were used for the analysis and converted to fasta using the qiime (Caporaso et al., 2010) “convert_fastaqual_fastq” function. The resulting fasta files were quality checked and annotated using the SILVA NGS pipeline (https://www.arb-silva.de/ngs/) (Glockner et al., 2017) using SILVA release version 123 with default settings (ambiguity and homopolymers 2%, OTU clustering 97%, min seq. quality 30%, min length 200 bp, min align. identity 50%). SILVAngs data analysis service uses the SILVA Incremental Aligner (SINA) and to remove contaminations of the dataset with non-rRNA sequences and removes sequences with ambiguity and homopolymers (max. 2%). After the quality control identical reads were dereplicated to operational taxonomic units (OTUs), on a per sample basis, and the reference read of each OTU is classified. Dereplication and clustering is based on the cd-hit-est version 3.1.2 (Li and Godzik, 2006). The clustering was performed with a minimum of 97% sequence identity to each other (pairwise distance and single linkage clustering). For each OTU, the longest read is used as a reference of this cluster for taxonomic classification by BLAST (version 2.2.28+) in combination with the SILVA SSURef dataset (release 123). The resulting classification of the reference sequence of a cluster is mapped to all members of the respective cluster as well as their replicates. Best BLAST hits were only accepted if they had a (sequence identity + alignment coverage) / 2 ≥ 93 or otherwise defined as unclassified. The quality management rejected 5,112,963 reads, 5,927,565 reads were classified in 1,200 bacterial genera (OTUs), and for 55,966 reads had no relative sequence were found.

OTU counts based on genus level rank classification were sum-normalized. Explicet (Robertson et al., 2013) was used for providing bootstraped-based subsampling for normalizing OTU counts in the bacterial richness analysis. For this analysis four samples with less than 19,360 reads were excluded. The difference in the bacterial community composition were visualized through non-metric multidimensional scaling (NMDS) plots using Bray-Curtis dissimilarity indices based on genus rank classification. We used the software package PAST (Hammer et al., 2008) to analyze differences between OTU compositions based on the analysis of similarities (ANOSIM) and a Tukey's pairwise test to calculate differences between the number of OTUs between the samples. A linear discriminant analysis (LDA) effect size (LEfSe) analysis (Segata et al., 2011) was performed to determine bacterial groups that are significantly enriched between samples using the “One against all” strategy for multi-class analysis (http://huttenhower.sph.harvard.edu/galaxy/). LEfSe uses a non-parametric test that couples standard tests for statistical significance with additional tests encoding biological consistency and effect relevance. Due to the relatively limited number of samples we used a *p*-value of 0.01 for significance.

### Data storage

The raw sequencing fastq files were submitted to European Nucleotide Archive under the primary accession number PRJEB23675.

## Results

### Patient materials /cohorts

The study population comprised 18 patients. 15 patients had CRS. Of those, five suffered from unilateral purulent maxillary CRS, five had CRSsNP and five had CRSwNP, respectively. Three patients undergoing surgery for an orbital tumor, orbital decompression and a posttraumatic mucocele, respectively, served as healthy controls. The distribution of gender and age is shown in Table 1.

**Table 1:** Distribution of gender, age and patient type of the study population.

**GENDER DISTRIBUTION**						
**Gender**	**Female**		**Male**			
Number of patients	10		8			
**AGE DISTRIBUTION**						
**Range Age**	**Number of patients**	**Average age**	**Unilateral purulent maxillary CRS**	**CRSsNP**	**CRSwNP**	**Healthy control group**
0–12 years	0	0				
13–21 years	2	18		2		
21–29 years	4	23.5	1	3		
30–50 years	5	42.6	3		1	1
50–60 years	4	52.2			4	
60–70 years	1	64	1			
>70 years	2	77				2

No eosinophilia (proportion of eosinophilic granulocytes <5%) was noted in all patients with CRSsNP and in four Patients with unilateral purulent maxillary CRS. One patient with unilateral purulent maxillary CRS and the five patients in the CRSwNP group had eosinophilia (>5%).

### Species identification from culture experiments

Although some of the species were only detected in one or two tissue samples, no systematic differences of the cultured species (i.e., significant difference in the number of cultured species or certain species that would have been cultured only from one specific location) were detected between the three distinct locations of tissue sampling. Therefore, the three tissue biopsies of each patient were analyzed together, and all detected species were counted no matter whether a species was detected in all three or only in one or two of the tissue samples.

Similarly, in this small cohort, no difference of cultured species was detected whether swabs were taken preoperatively from the middle meatus or intraoperatively from the opened sinuses. Therefore, the two swabs taken of each patient were analyzed together, and all detected species were counted no matter whether a species was detected in both, or only in one of the two swabs. Species identified in cultures from swabs and mucosal biopsies are listed in Table 2.

**Table 2:** Species identified from culture experiments within the different patient types.

**Unilateral purulent maxillary CRS**		**CRS with nasal polyps (CRSwNP)**		**CRS without nasal polyps (CRSsNP)**		**Healthy sinus mucosa**	
**Swab-cultures**	**Biopsie-cultures**	**Swab-cultures**	**Biopsie-cultures**	**Swab-cultures**	**Biopsie-cultures**	**Swab-cultures**	**Biopsie-cultures**
Enterobacteria	Enterobacteria	Enterobacteria	Enterobacteria	Enterobacteria	Enterobacteria	Enterobacteria	Enterobacteria
*C. braakii* (1)	*C. braakii* (1)	*E. cloacae* (1)	*E. cloacae* (1)	*E. coli* (1)	*E. coli* (1)	*K. pneumoniae* (1)	
*C. youngae* (1)	*C. youngae* (1)	*P. mirabilis* (1)	*P. mirabilis* (1)	*S. marcescens* (1)		*K. oxytoca* (1)	*K. oxytoca* (1)
*K. pneumoniae* (1)	*K. pneumoniae* (1)	*K. oxytoca* (1)	*K. oxytoca* (1)				
*R. gilardii* (1)	*R. gilardii* (1)	*K. pneumoniae* (1)					
		*C. koseri* (1)	*C. koseri* (1)				
							
Staphylococci	Staphylococci	Staphylococci	Staphylococci	Staphylococci	Staphylococci	Staphylococci	Staphylococci
*S. aureus* (1)		*S. epidermidis* (5)	*S. epidermidis* (5)	*S. epidermidis* (5)	*S. epidermidis* (5)	*S. aureus* (2)	*S. aureus* (2)
*S. epidermidis* (4)	*S. epidermidis* (4)	*S. pasteuri* (1)		*S. aureus* (1)	S. aureus (1)	*S. epidermidis* (3)	*S. epidermidis* (2)
*S. capitis* (2)	*S. capitis* (1)	*S. hominis* (1)	*S. hominis* (1)	*R. mucilanginosa* (1)		*S. capitis* (2)	*S. capitis* (1)
		*S. capitis* (3)			*S. capitis* (1)	*S. lugdunensis* (1)	
		*S. aureus* (2)					
							
Streptococci	Streptococci	Streptococci	Streptococci	Streptococci	Streptococci	Streptococci	Streptococci
*S. anginosus* (1)	*S. anginosus* (1)	Viridans streptococci (1)		Viridans streptococci (1)	Viridans streptococci (1)		
Viridans streptococci (1)	Viridans streptococci (1)						
Anaerobic bacteria	Anaerobic bacteria	Anaerobic bacteria	Anaerobic bacteria	Anaerobic bacteria	Anaerobic bacteria	Anaerobic bacteria	Anaerobic bacteria
*P. granulosum* (1)	*P. granulosum* (1)	*F. magna* (1)	*F. magna* (1)	*P. acnes* (5)	*P. acnes* (5)	*P. acnes* (3)	*P. acnes* (3)
*P. acnes* (3)	*P. acnes* (3)	*P. acnes* (5)	*P. acnes* (5)	*F. magna* (1)	*F. magna* (1)	*Bifidobacterium* spp. (1)	*Bifidobacterium* spp. (1)
P. oris (1)	*P. oris* (1)	*P. granulosum* (1)	*P. granulosum* (1)	*P. granulosum* (1)	*P. granulosum* (1)		*P. avidum* (1)
*F. nucleatum* (2)	*F. nucleatum* (2)	*B. urealyticus* (1)					
*P. micra* (1)	*P. micra* (1)	*P. avidum* (1)	*P. avidum* (1)				
*P. avidum* (1)							
*Bifidobacterium* sp. (1)	Bifidobacterium sp. (1)						
Coryneforme bacteria	Coryneforme bacteria	Coryneforme bacteria	Coryneforme bacteria	Coryneforme bacteria	Coryneforme bacteria	Coryneforme bacteria	Coryneforme bacteria
*Corynebacterium* spp. (2)	*Corynebacterium* spp. (1)	*Corynebacterium* spp. (4)	*Corynebacterium* spp. (4)	*Corynebacterium* spp. (3)	*Corynebacterium* spp. (2)	*Corynebacterium* spp. (3)	*Corynebacterium* spp. (1)
Haemophilus	Haemophilus	Haemophilus	Haemophilus	Haemophilus	Haemophilus	Haemophilus	Haemophilus
		*H. parainfluenzae* (1)		*H. influenzae* (1)	*H. influenzae* (1)		
				*H. parainfluenzae* (1)		Lysinibacillus	Lysinibacillus
						*Lysinibacillus* spp. (1)	*Lysinibacillus* spp. (1)

*The table shows the bacteria identified in the swab- and biopsie-cultures, respectively. The number in brackets reflects the number of patients found with the named/corresponding species in the investigated patient type*.

**Table 3:** Overview of the sampled tissues with age and gender of each patient. “m”: male, “f”: female.

**Patient number**	**Age**	**Gender**	**Patient type**	**Tissue**	**Probe-number**
5	34	m	Unilateral purulent maxillary CRS	Processus uncinatus	5A
				Bulla ethmoidales	5B
				Maxillary sinus	5C
6	25	f	Unilateral purulent maxillary CRS	Processus uncinatus	6A
				Bulla ethmoidales	6B
				Maxillary sinus	6C
7	56	m	CRSwNP	Processus uncinatus	7A
				Bulla ethmoidales	7B
				Nasal polyp	7C
8	25	f	CRSsNP	Processus uncinatus	8A
				Bulla ethmoidales	8B
				Recessus frontalis	8C
9	17	f	CRSsNP	Processus uncinatus	9A
				Bulla ethmoidales	9B
				Recessus frontalis	9C
10	23	m	CRSsNP	Processus uncinatus	10A
				Bulla ethmoidales	10B
11	21	m	CRSsNP	Processus uncinatus	11A
				Ethmoidbone	11B
				Maxillary sinus	11C
12	19	m	CRSsNP	Processus uncinatus	12A
				Bulla ethmoidales	12B
				Maxillary sinus	12C
13	52	m	CRSwNP	Processus uncinatus	13A
				Bulla ethmoidales	13B
				Maxillary sinus	13C
14	47	f	CRSwNP	Processus uncinatus	14A
				Nasal polyp	14B
				Ethmoidbone polyp	14C
15	44	f	Healthy control	Processus uncinatus	15A
				Bulla ethmoidales	15B
				Concha bullosa	15C
16	39	f	Unilateral purulent maxillary CRS	Processus uncinatus	16A
				Bulla ethmoidales	16B
				Maxillary sinus	16C
17	49	f	Unilateral purulent maxillary CRS	Processus uncinatus	17A
				Bulla ethmoidales	17B
				Maxillary sinus	17C
18	80	m	Healthy control	Bulla ethmoidales	18A
				Maxillary sinus	18B
				Basic lamella	18C
19	51	f	CRSwNP	Processus uncinatus	19A
				Concha bullosa	19B
				Ethmoidbone polyp	19C
20	51	m	CRSwNP	Processus uncinatus	20A
				Concha bullosa	20B
				Nasal polyp	20C
21	64	f	Unilateral purulent maxillary CRS	Processus uncinatus	21A
				Bulla ethmoidales	21B
				Concha bullosa	21C
22	74	f	Healthy control	Processus uncinatus	22A
				Bulla ethmoidales	22B
				Nasenmuschel	22C

Both, aerobic and anaerobic bacteria were found with each sampling method in all patient groups. From tissue samples 4.2 ± 1.2 (mean ± sd) distinct species per patient were cultured, from swabs 5.4 ± 1.6 (*p* = 0.017, ANOVA on ranks). Species that in certain patients were detected from swabs, but not from tissue samples were *Staphylococcus capitis* (5 cases), *Corynebacterium* spp. (4), *Staphylococcus aureus* (3), *Klebsiella pneumoniae* (2), *Haemophilus parainfluenzae* (2), *Propionibacterium avidum, Staphylococcus pasteuri, Bacteroides urealyticus, Serratia marcescens, Staphylococcus lugdunensis*, and *Staphylococcus epidermidis*. Of those, *K. pneumoniae, H. parainfluenzae, S. pasteuri, B. urealyticus, S. marcescens*, and *S. lugdunensis* were only cultured from swabs, but never from tissue samples. Only in two patients, species were identified that were only present in the tissue sample, one *P. avidum* and one *S. capitis*.

In all patient groups, *Enterobacteria, Staphylococci*, coryneform bacteria and *Propionibacteria* were detected. Viridans streptococci were also found with exception of the healthy control group. *Haemophilus* were only found in probes of patients with CRSsNP and CRSwNP, while *Lysinibacillus* could only be detected in one patient in the healthy control group.

Looking at species level, *S. aureus, S. capitis, S. epidermidis*, Corynebacterium spp. and *Propionibacterium acnes* were observed in all groups. *P. acnes, S. epidermidis* and coryneform bacteria were detected in most (3–5 of each group) patient samples, whereas *S. capitis* and *S. aureus* could only be identified in one or two patient-samples of each group.

### Cultivation independent investigation of the bacterial community composition

**Figure 1:**
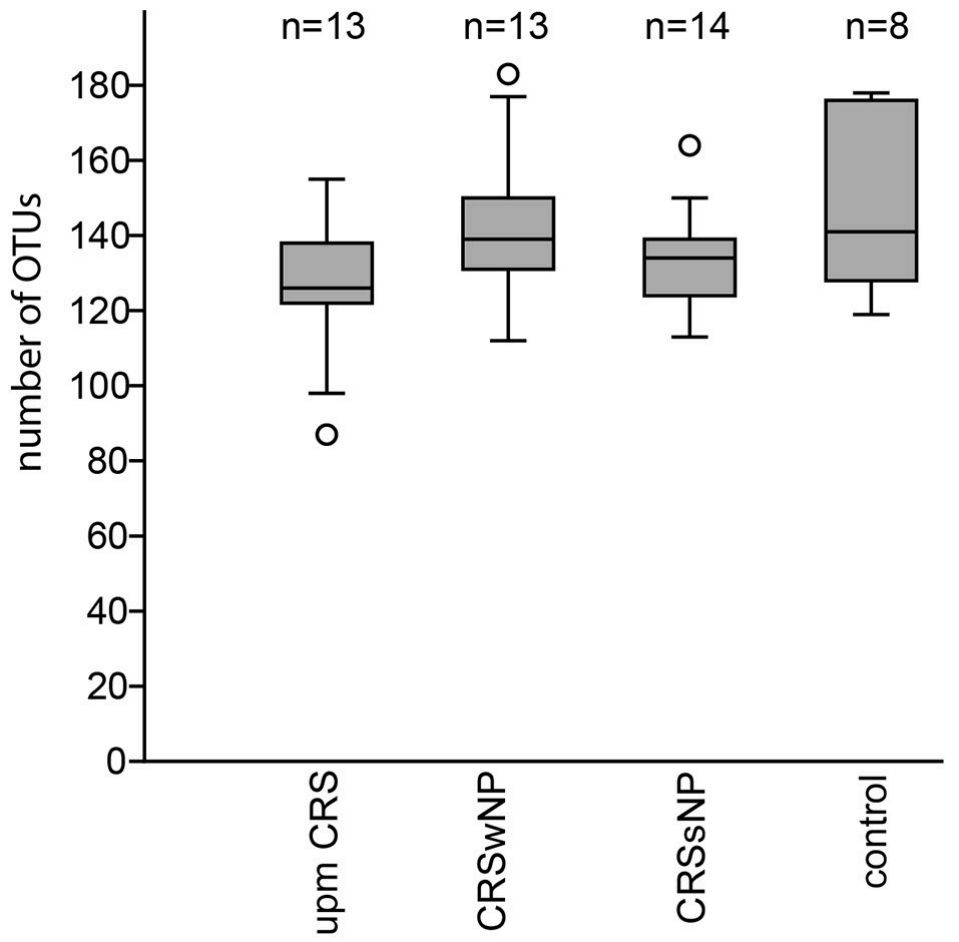
Boxplot of the rarefied, bootstrapped number of taxa found for unilateral purulent maxillary CRS (upm CRS), CRS with nasal polyps (CRSwNP), and CRS without nasal polyps CRSsNP. The boxplot shows the 25–75% quartiles; the median is indicated by the horizontal line inside the box. The largest data points <1.5 times the box height (“upper-inner fence”) are shown with short horizontal lines and similarly below the box. Values outside the inner fences are shown as circles.

**Figure 2:**
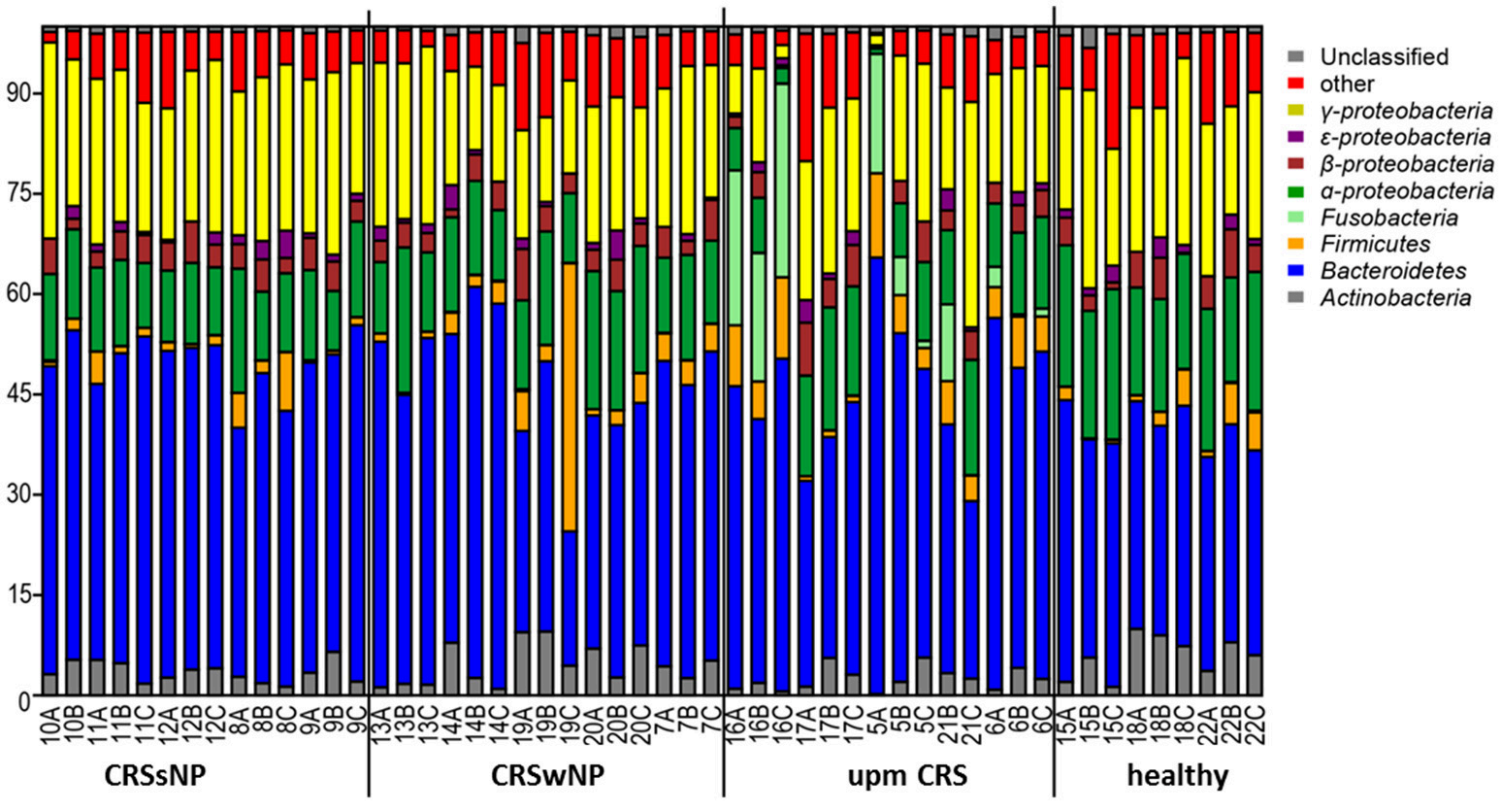
Bacterial community composition is shown on phylum/class level for unilateral purulent maxillary CRS (upm CRS), CRS with nasal polyps (CRSwNP), and CRS without nasal polyps (CRSsNP). The samples were ordered based on the different CRS types. For detailed information of each sample refer to Table 3.

**Figure 3:**
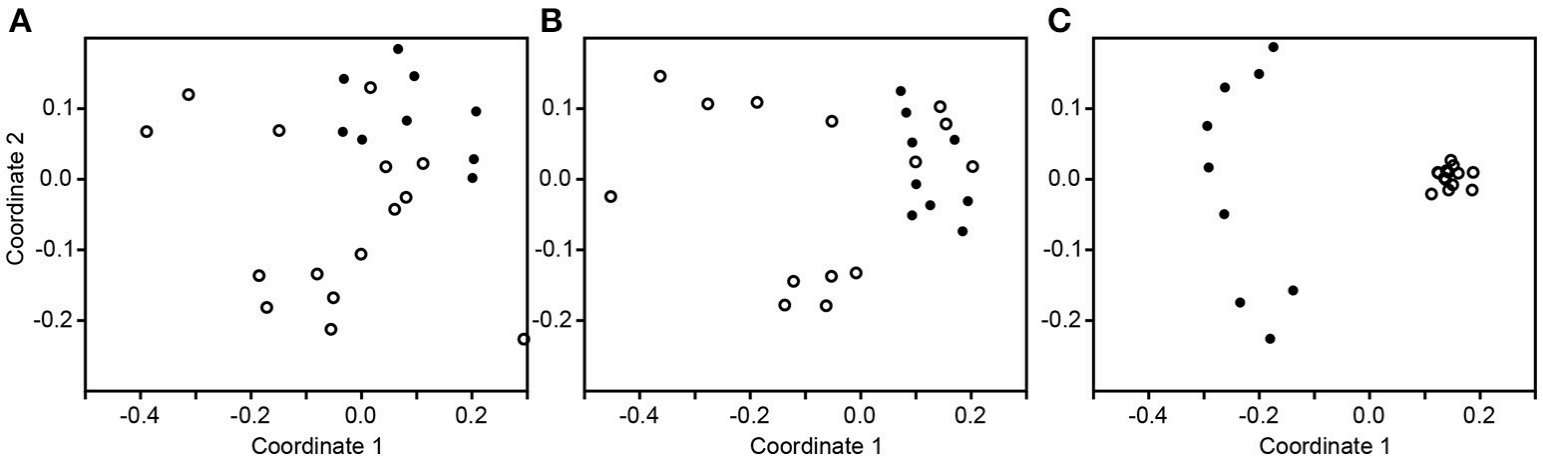
Non-metric multidimensional scaling plots based on Bray-Curtis dissimilarity of the healthy samples (dot) compared to the inflamed samples (circle) in unilateral purulent maxillary CRS **(A)**, diffuse CRS with nasal polyps **(B)**, and without nasal polyps **(C)**.

**Figure 4:**
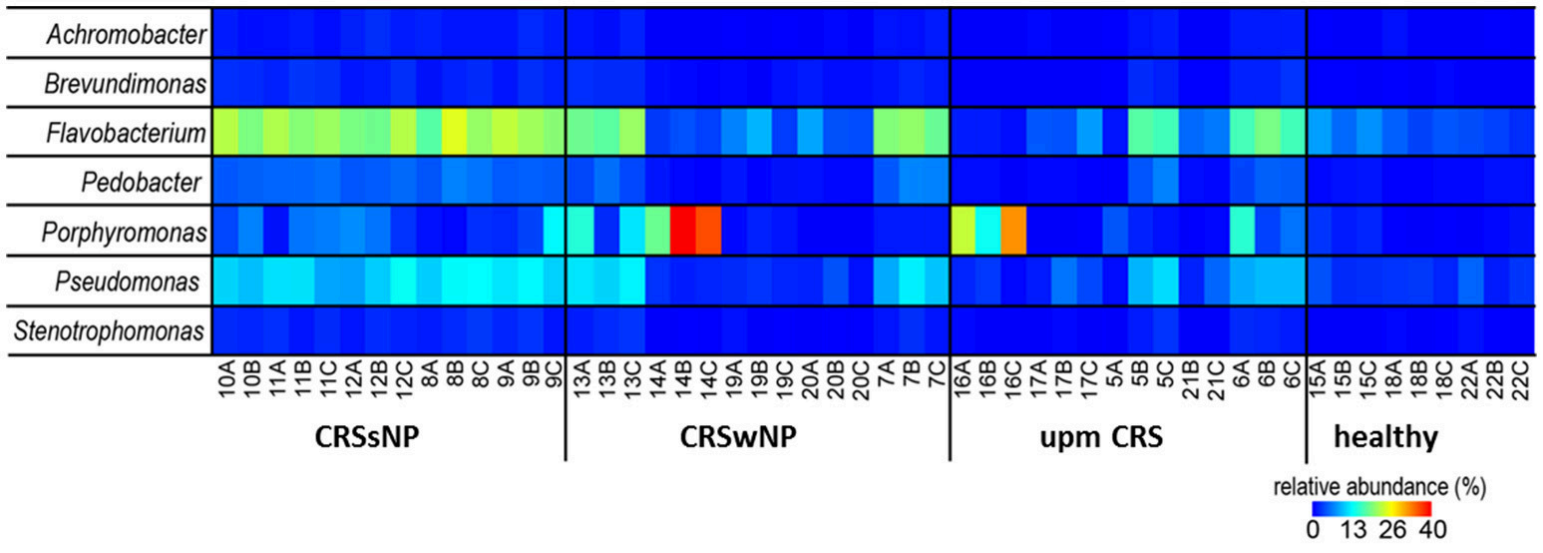
Relative abundance of the significantly enriched bacterial genera in CRS without nasal polyps identified by the LefSe analysis. The samples were ordered based on the different CRS types: unilateral purulent maxillary CRS (upm CRS), CRS with nasal polyps (CRSwNP), and CRS without nasal polyps (CRSsNP). For detailed information of each sample refer to Table 3.

The subsampled number of bacterial taxa detected in the cultivation independent investigation was compared between CRSwNP, CRSsNP, and unilateral purulent maxillary CRS patients. Pooling all different tissues from each CRS type revealed an insignificantly lower number of bacterial taxa for the CRS compared to the healthy controls (Figure 1). On phylum/class level the bacterial community composition of unilateral purulent maxillary CRS consisted in several samples also of *Fusobacteria* (Figure 2). The bacterial community composition of the different tissues did not reveal any pattern (Figures 2, Figure S1). Therefore, we pooled the different tissues and compared healthy samples with the sample of the patients. Considering all tissue types no clear pattern in the NMDS plots were visible for unilateral purulent maxillary CRS and CRSwNP (Figures 3A,B). The *p*-values in the ANOSIM test (*p* = 0.04) were also not significant. In contrast to unilateral purulent maxillary CRS and CRSwNP, the bacterial community of patients with CRSsNP was significantly (*p* < 0.01) separated from the bacterial community of healthy subjects (Figure 3C). The subsequent LefSe analysis indicated that the bacterial genera *Flavobacteria, Pedobacter, Stenotrophomonas, Pseudomonas, Porphyromonas, Brevundimonas*, and *Achromobacter* had a significantly higher abundance in these samples (Figure 4). Similar to the cultivation-dependent analysis, the tissue type showed again no trend for the bacterial community composition.

### Comparison of the cultivation independent and cultivation approaches

**Figure 5:**
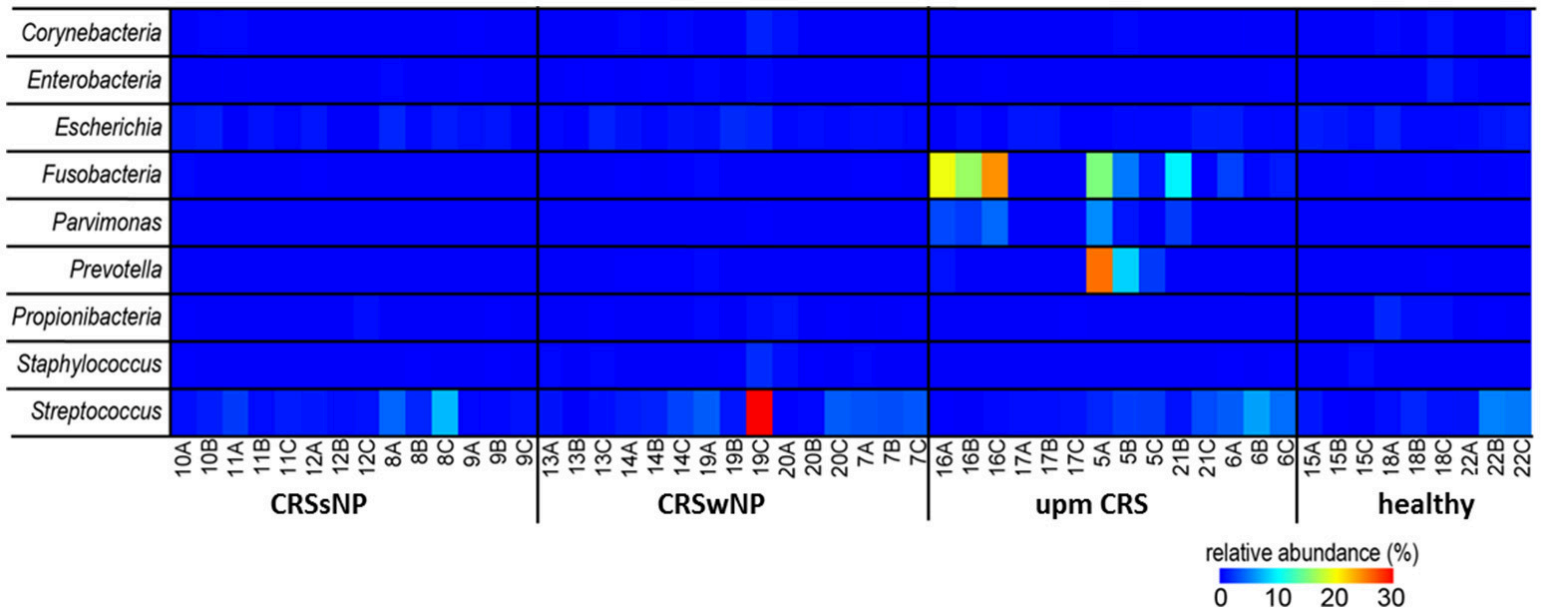
Comparison of the successfully isolated bacteria (see Table 2) and their relative abundance in the cultivation independent analysis. The samples were ordered based on the different CRS types: unilateral purulent maxillary CRS (upm CRS), CRS with nasal polyps (CRSwNP), and CRS without nasal polyps (CRSsNP). For detailed information of each sample refer to Table 3.

Since many typically CRS associated bacterial taxa were found in the cultivation dependent analysis, we investigated their presence in the dataset from the cultivation independent analysis. This revealed a strong discrepancy between the bacteria found in culture and those found based on cultivation independent investigation. The genera *Bifidobacterium, Citrobacter, Finegoldia, Klebsiella, Haemophilus, Roseomonas, Lysinibacillus*, and *Proteus* were found in very low abundances (average <0.05%) or not at all in the cultivation independent study (Figure 5). Most of the other bacterial genera in the cultivation based analysis—in particular the genera *Staphylococcus* (range: 0–2.1%), *Corynebacterium* (range: 0–1.6%) and *Propionibacterium* (range: 0–1.9%) that were cultivated from almost all of the patients—were only present in low abundances in the cultivation-independent analysis. *Escherichia* and *Streptococcus* were found in all samples, including healthy tissues. Only *Fusobacteria, Parvimonas*, and *Prevotella* that were found in the samples of two specific cases of unilateral purulent maxillary CRS by the cultivation were also found in the cultivation independent approach in high abundances.

## Discussion

### CRS-subgroups

CRSwNP and CRSsNP are the two major clinical phenotypes that also imply different therapeutic approaches with CRSwNP being more responsive to therapy with corticosteroids. In the western countries, the disease process in CRSwNP seems to be dominated by a pathologic host immune response with a Th2-skew and eosinophilic infiltration of the mucosa (Dennis et al., 2016). The immunologic cascades are increasingly well understood, which is currently leading to an endotyping of distinct types of CRS (CRSwNP) enabling targeted pharmacotherapy of the pathologic immunologic process. Increasing levels of IL-5 and staphylococcal superantigen production seem to increase the likelihood of a phenotype of CRSwNP and comorbid asthma (Tomassen et al., 2016). For CRSsNP the immunologic patterns are less clear and external factors such as presence of bacteria might play a more significant role (Fokkens et al., 2012). This idea is supported by recent evidence that non-sinusitis-related antibiotic therapy is a risk factor for a development of CRSsNP, but not of CRSwNP (Maxfield et al., 2016).

Among the patients with CRSsNP we decided to distinguish between those with diffuse disease affecting both sides and all sinuses, and those where only one side is affected with a completely healthy contralateral side. We hypothesize that in unilateral cases either an undetected odontogenic focus (Matsumoto et al., 2015) or a localized obstruction of the ostiomeatal complex might significantly contribute to the disease process, possibly leading to a distinct contribution of bacteria to the disease process. Therefore, three groups of patients were investigated: CRSwNP, diffuse CRSsNP and unilateral maxillary CRS (without evident odontogenic focus). Selection of the patients was based on the pre- and intraoperative endoscopic findings. All patients in the CRSwNP group had evident polypoid masses, two of which were recurrent cases after previous sinus surgery. For the CRSsNP group, patients with signs of mucosal edema were excluded, because early CRSwNP can appear very similar to CRSsNP (Dennis et al., 2016).

### Culture experiments

The interest in the microbiology of CRS is clearly reflected by the increase of publications about this topic from about 20 per year in the early 90's to 80–120 per year between 2005 and 2015. It is now commonly accepted that both diseased and healthy sinuses are inhabited by a variety of microbiota including species regarded as potentially pathogenic (Hoggard et al., 2017). Until 2012 bacterial culture was the basis of the published studies and the focus was to identify single bacterial species being causative for the disease and a possible target for antimicrobial therapy. Brook (2016) recently provided a review about the trends that can be derived from this plethora of studies. Some colonizing bacteria such as *Propionibacteria, Corynebacteria* and coagulase-negative *Staphylococcus* (although interpreted by Brook as frequent contamination) can be frequently cultured from healthy and diseased sinus mucosa. While acute Rhinosinusitis (ARS) is associated with aerobe pathogens such as *Streptococcus pneumoniae, Haemophilus influenza*, and *Moraxella catarrhalis*, in CRS the probability to cultivate certain potentially pathogenic anaerobes such as *Fusobacterium nucleatum, Prevotella, Porphyromonas*, and *Peptostreptococcus* spp. increases. *S. aureus* is more frequently cultured from CRS compared to ARS, but it is cultured from healthy mucosa in comparable frequency. Gramnegative aerobes such as *Pseudomonas aeruginosa, K. pneumoniae, Escherichia coli, Enterobacter* spp., and *Proteus mirabilis* are more often cultivated after multiple courses of antibiotic therapy and/or previous sinus surgery. No difference between CRSwNP and CRSsNP could be detected (Brook, 2016). Accordingly, in our study *P. acnes, Corynebacterium* spp. and *S. epidermidis* were cultured from most samples of both healthy and diseased patients. These species were only missing in two cases of unilateral maxillary sinusitis where *F. nucleatum* in one case and *Streptococcus anginosus* in the other case were cultured in massive quantities. *Enterobacteriaceae* and *S. aureus* were cultured from few patients of all groups, while *Haemophilus* spp. and *Streptococcus viridans* were only cultured from diseased patients.

In this study, both swabs (middle meatus and intraoperatively) and tissue samples from three separate locations of sinus mucosa (mostly uncinate process, bulla ethmoidalis and medial wall of the maxillary sinus) were brought into culture. While we did not detect significant differences between the three tissue samples and the two swabs, respectively, significantly more distinct species were cultured from the swabs compared to the tissue samples. Considering the significant difference despite our relatively small sample size and the fact that other authors also described the phenomenon (Niederfuhr et al., 2009) corroborates an important difference of the bacterial flora of middle meatus secretions and sinus mucosa, at least for the cultivable part of it. The immune barrier hypothesis states that a progressive loss of mechanical and/or immunological barrier function facilitates further exposure to environmental stimuli such as bacteria (Kern et al., 2008; Hoggard et al., 2017). We hypothesized that the bacteriology of secretions might be of rather transient nature while the species invading the mucosa might better reflect the chronic long-term alterations in the diseased patients and, therefore, used tissue specimens for the microbiome analysis.

The results of microbiome analyses question the meaningfulness of culture results in general. Which species grow in culture rather depends on their ability to grow on the used agar medium (Boase et al., 2013) than on their actual abundance or their significance for the disease process. More intensive culture efforts detect 4–5 times more different species per patient compared to standard cultures (Kaspar et al., 2016), while culture-independent sequencing-based studies detect up to an order of magnitude more distinct taxa (Boase et al., 2013; Hauser et al., 2015). Since in clinical practice culturing swabs is currently the gold standard for assessment of bacteria one aim of this study was to test, whether standard culture can predict at least certain aspects of the microbiome.

### Microbiome analysis

Microbiome analyses of sinunasal samples with sequencing techniques have emerged since 2012 and have already revolutionized our view of microbiota in both healthy and diseased states. Only recently the first comprehensive reviews (Ramakrishnan et al., 2016; Hoggard et al., 2017) have been published, and despite of many contradictory statements in the different studies some common trends emerge. Less diversity in the microbial community rather than an increased overall bacterial load seems to characterize CRS compared to the healthy state with fewer consensuses about specific genera indicative of disease (Ramakrishnan et al., 2016; Hoggard et al., 2017).

In our study tissue samples of three distinct locations were analyzed. One biopsy was taken of the first accessible tissue with broad contact to the nasal cavity, either the uncinate process in primary cases or the first accessible polyps in cases of recurrent CRSwNP. Two more tissue samples were taken from regions deep in the sinuses. We did not detect any systematic pattern in the composition of the microbial community within these locations. This is to our knowledge the first report about missing spatial organization of the microbiome from tissue samples. Our results agree with studies showing that swabs of the middle meatus are representative of deeper sinus regions (Ramakrishnan et al., 2017) and the sphenoethmoid recess (Yan et al., 2013). Swabs from the inferior meatus (Lal et al., 2017) or nares (Yan et al., 2013) have shown a distinct composition of the bacterial community.

A major difference of our study compared to most others is that tissue samples were examined instead of swabs or brush samples. Two previous studies comparing microbiome analyses from swabs and tissue samples gained contradictory results. While Bassiouni et al. did not detect a significant difference in samples of 6 CRS patients (Bassiouni et al., 2015), Kim et al. found significant differences in the composition of bacterial communities in a study of 9 CRS patients (Kim et al., 2015). Kim et al. speculated that the bacteria on the surface seed the underlying tissue via the damaged epithelium in CRS patients, which over time develops into a distinct bacterial community, which might be more characteristic of the disease process.

In our analysis the CRS samples were slightly but insignificantly lower in bacterial richness compared to healthy subjects (Figure 1). This indicates that not all cases of CRS are characterized by a lower overall bacterial richness and studies with more patients are necessary to understand investigate this effect.

The differences in the microbiome were also insignificant for CRSwNP and unilateral purulent maxillary CRS. This also reflects that the bacterial communities among CRS patients differ and may be even comparable to bacterial communities of healthy patients (Figures 2, 3). Also in this case, significant trends may become visible with larger sampling cohorts.

Nevertheless, despite our relatively small sample size, patients with CRSsNP showed a very clear and significant difference in their bacterial community composition compared to healthy subjects. Among the bacterial groups that were significantly enriched in these patients were *Flavobacteria, Pedobacter, Stenotrophomonas, Pseudomonas, Porphyromonas, Brevundimonas*, and *Achromobacter* (Figure 4). This indicates that bacteria are more than a mere bystander but possibly play a role in the disease process of CRSsNP. Whether these bacteria act as direct drivers of the disease or just contribute to progression and exacerbation of the disease cannot be answered with the design of our study. The genera *Flavobacteria, Pedobacter, Brevundimonas*, and *Achromobacter* do not comprise relevant human pathogens. Members of the genus *Porphyromonas* can cause periodontitis and root canal abscesses (Nickles et al., 2016; Nobrega et al., 2016; Tomas et al., 2017) and were associated with CRS in CoNet analyses in a recent meta-analysis of the 16S rRNA sequencing data (Wagner Mackenzie et al., 2017). Members of the genera *Stenotrophomonas* and *Pseudomonas* are frequently involved in hospital acquired infections and have been already associated with CRS in many culture-based studies (Brook, 2016). Cope et al. have recently described four sub-groups of CRS that predicted the clinical phenotype. Each sub-group was defined by a specific pattern of bacterial co-colonization dominated by a pathogenic family, one of which belonged to the family *Pseudomonaceae* (Cope et al., 2017). A strain of *P. aeruginosa* isolated from a patient with CRS caused an acute infection in rabbits with a pervasive shift in the sinus microbiome that persisted despite histologic resolution (Cope et al., 2016). Consequently, the significance of *P. aeruginosa* for the development of CRS warrants further research.

Our results put up the question whether bacteria indeed play a more significant role in CRSsNP compared to CRSwNP, especially since some other studies did not show such a clear difference (Biswas et al., 2015; Ramakrishnan et al., 2015). By excluding unilateral cases of CRSsNP we might have more strictly defined this entity compared to other studies. By chance, all 5 cases of CRSsNP were primary cases of young patients below 30 years of age (Table 1) without tissue eosinophila, while in the CRSwNP group with 3 primary and 2 revision surgeries all had tissue eosinophilia. Furthermore, there are several observations supporting the hypothesis of a more significant role of bacteria in CRSsNP. Other studies have also demonstrated a clearer difference between healthy vs. CRSsNP compared to healthy vs. CRSwNP (Lal et al., 2017). The better response of CRSwNP to steroid therapy suggests a more significant role of the inflammatory process, while non-sinusitis-related antibiotic therapy is a risk factor for the development of CRSsNP, but not of CRSwNP (Maxfield et al., 2016), suggesting a more important role of bacteria. A most recent study that analyzed 59 CRS patients and 10 healthy controls with sophisticated statistical methods, though, could predict both phenotypes and immunologic pathways from microbiological clusters (Cope et al., 2017).

The species brought into culture were not necessarily abundant in the cultivation independent analysis. Only in two cases of unilateral maxillary CRS the genera *Fusobacterium, Prevotella*, and *Parvimonas* were found at high abundance in the cultivation independent analysis, while in parallel *F. nucleatum, Parvimonas micra*, and *Prevotalla oris*, were found in the cultivation based study in high quantities. These bacteria are typical members of the oral flora and regularly cultivated from chronic periodontitis (Nickles et al., 2016; Tomas et al., 2017). They are known as pathogens in acute root canal infections (Nobrega et al., 2016) and particularly *Fusobacteria* have a high virulence and are frequently involved in severe infections such as peritonsillar abscesses (Powell et al., 2013) and intracranial sinugenic complications (Gallagher et al., 1998). A 16S rRNA based sequencing analysis of acute enteric infections revealed a high abundance of the *Enterobacter* that comprised the cultivated pathogens of between 20% and 99% in 61% of the cases (Singh et al., 2015). Thus, an unusual high abundance of a pathogen-related taxa detected in sequencing analyses seems to be characteristic of classic acute bacterial infections.

These observations allow for some speculation about future directions for microbiological diagnostics of rhinosinusitis in clinical practice. It seems plausible that the infection with *Fusobacterium, Prevotella*, and *Parvimonas* in the two specific cases of unilateral purulent maxillary CRS represented persistent classic infections with single pathogenic bacteria of odontogenic origin. In the two patients bacterial taxa belonging to highly abundant genera in the 16S rRNA sequencing analysis could also be cultivated, thus cultivating swabs served its task to identify a potentially causative pathogen and thus to guide antibiotic therapy. In many other cases of our study, putative pathogens were cultured that were not abundant in the microbiome. Whether these cultivated bacteria played a role for the disease process seems questionable, but they might have prompted an antibiotic treatment in the clinical situation that may not have the expected effect. Consequently, 16S rRNA sequencing might help to interpret the results of classic bacterial culture. Furthermore, sequencing techniques might allow for more rapid diagnosis of relevant bacteria involved in acute infections. This seems to work already for meningitis (Srinivasan et al., 2012) and bloodstream infections (Su et al., 2015), where the analyzed materials (cerebrospinal fluid and blood) are sterile in the healthy state. With increasing knowledge about the normal spectrum of abundance of the bacterial community composition comprising typical sinunasal pathogens it should be possible to define thresholds above which an acute bacterial infection by a member of this bacterial community can be suspected. Future studies of the microbiome of acute rhinosinusitis and its predictive value for the success of an antibiotic therapy are warranted.

## Author contributions

BO acts as the submissions guarantor. BO, AP, KK, and BK designed the study. BO, TS, AO, and EG collected the samples and performed the clinical part of the study. KK performed the microbiologic workup of the samples. DH performed the 16S rRNA gene analysis. DH, KK, BO, and BK analyzed the data. BO, KK, BK, and DH wrote the manuscript. All authors approved the definitive version of the manuscript.

### Conflict of interest statement

The authors declare that the research was conducted in the absence of any commercial or financial relationships that could be construed as a potential conflict of interest.
