# Supplementary material for: Microbiome and Culture Based Analysis of Chronic Rhinosinusitis Compared to Healthy Sinus Mucosa
Source: Front Microbiol. 2018 Apr 17;9:643. doi: 10.3389/fmicb.2018.00643 (PMC5932350; doi:10.3389/fmicb.2018.00643)
Supplement: Supplementary file 1 [file Image1.PDF]

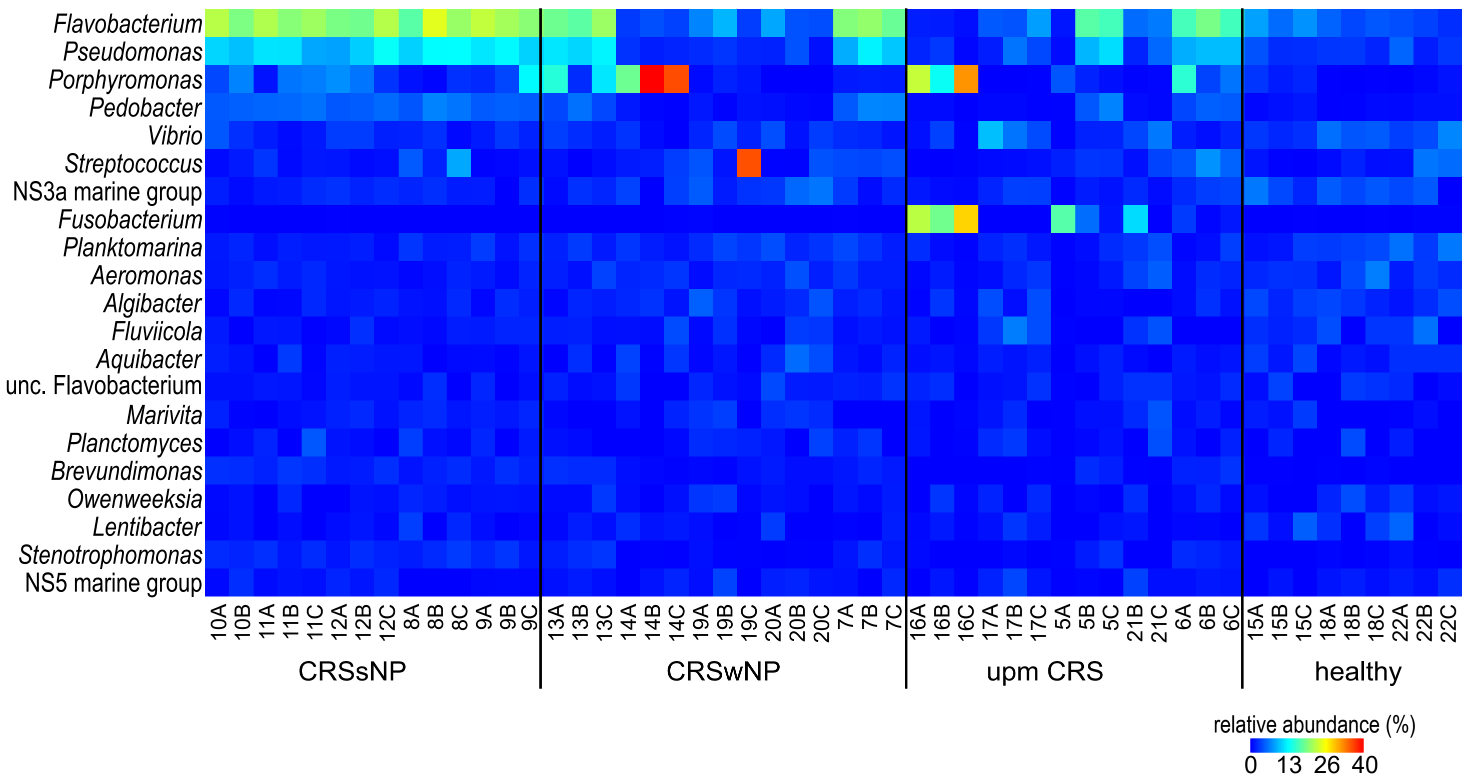


SFig 1. Relative abundance of the 1% most abundant bacterial genera. The samples were ordered based on the different CRS types. For the detailed information of each sample see table 3.
